# The analysis of knee joint loading during drop landing from different heights and under different instruction sets in healthy males

**DOI:** 10.1186/s40798-016-0072-x

**Published:** 2017-01-18

**Authors:** Dmitry Verniba, Jason D. Vescovi, David A. Hood, William H. Gage

**Affiliations:** 1 Orthopaedic Neuromechanics Laboratory, Sherman Health Science Research Centre, York University, Toronto, Ontario Canada; 2 Muscle Health Research Centre, York University, Toronto, Ontario Canada; 3 School of Kinesiology and Health Science, York University, Toronto, Ontario Canada

**Keywords:** Biomechanics, Drop landing, Physical activity, Force, Moment

## Abstract

**Background:**

Mechanical loading during exercise has been shown to promote tissue remodeling. Safe and accessible exercise may be beneficial to populations at risk of diminished bone and joint health. We examined the effect of drop height and instruction on knee loading during a drop-landing task and proposed a task that makes use of drop heights that may be appropriate for rehabilitation purposes and functional in daily life to examine transient knee joint loads.

**Methods:**

Twenty males (22.0 ± 2.8 years) performed drop landings from 22 cm (low) and 44 cm (high) heights, each under three instructions: “land naturally” (natural), “softly” (soft), and “stiffly” (stiff). Knee compression force and external flexion moment were estimated using three-dimensional inverse dynamics and normalized to body mass.

**Results:**

Peak knee compression force was larger (*p* < 0.001) for high (17.8 ± 0.63 N/kg) than low (14.8 ± 0.61 N/kg) heights. There was an increase (*p* < 0.001) in the knee compression force across soft (11.8 ± 0.40 N/kg), natural (17.0 ± 0.62 N/kg), and stiff (20.2 ± 0.67 N/kg) instructions. Peak knee flexion moment in high-natural (2.12 ± 0.08 Nm/kg) was larger (*p* < 0.001) than in high-soft (1.88 ± 0.08 Nm/kg), but lower than in high-stiff (2.23 ± 0.08 Nm/kg). No differences in peak knee flexion moment were observed across instructions for the low height.

**Conclusions:**

We propose a drop-landing task that creates a scalable increase in knee compression loading. The absence of increased knee flexion moment with drop from the low height, compared to high, suggests that individuals could perform the task without incremental risk of knee injury. This task could be used in future studies to examine the effect of acute bouts of mechanical loading on bone and cartilage metabolism.

## Key Points

- The current drop-landing task creates an increase in knee compression loading with instruction to land stiffly.
- The absence of increased knee flexion moment during landing from a 22 cm height (household stair) suggests that individuals could perform the task without an increased risk of knee joint injury.
- The current task could be used in future studies to examine the effect of acute bouts of mechanical loading on bone and cartilage metabolism, and bone and joint health.

## Background

The effects of physical activity and resulting mechanical loads on bone health and articular cartilage integrity have implications for many groups including military [1], athletes [2], postmenopausal women [3], children [4], older adults [5], and clinical populations such as individuals with osteoarthritis [6] and individuals with stroke [7]. Researchers have reported positive, site-specific adaptations to bone mineral density in response to chronic exposure to activities associated with high mechanical loads (volleyball, basketball) compared to other types of activities (running, swimming) [8, 9]. In contrast, prolonged absence of mechanical loading (bed rest) leads to disuse osteoporosis [10]. Similarly, the health of articular cartilage is maintained through natural formation and degradation processes [11]. Short-term and acute responses of skeletal and articular cartilage metabolism to physical activity have been reported [12–14]; however, findings across studies are difficult to compare because the types of activities and research methodology are not standardized [14]. Traditionally, animal models have been used to examine the effects of mechanical loading on bone and articular cartilage [15, 16]; however, a comparable and standardized model to assess the acute response of bone and cartilage turnover to various levels of mechanical loading has not yet been clearly defined in humans [17]. To improve clinical utility of exercise that may result in a net formation of bone and cartilage in humans, it is important to have a standardized approach that allows researchers to examine responses, to quantify doses of mechanical loads, and to determine if a dose-response exists [14].

In developing an exercise, it is critical that risk of injury is not unduly elevated. It has been suggested that most acute knee joint soft tissue injuries are non-contact in nature, that is, the injury results from a person’s own movement and not a contact with an object or a person [18]. Further, it has been shown that excessive sagittal knee joint moment during landing may result in ligament injury [19, 20]. Instructions related to how to execute the landing have been shown to affect joint loading during drop landing [21–23], but no research has examined the influence of instruction set on loading during drop landings from a relatively low height. Researchers have previously described lower limb joint loading during drop landings from relatively large heights (e.g., >59 cm) in younger people [24], but these heights are prohibitive for individuals recovering from a joint injury or those with existing degenerative joint conditions.

The purpose of the current study was (1) to explore a drop-landing task that makes use of drop heights that may be more appropriate for rehabilitation purposes and are functional in daily life and (2) to examine transient knee joint loads during the drop-landing response to examine the effect of drop height and instruction cues on knee joint loads, with a focus to evaluating the potential for incremental injury risk during the task. The drop-landing task may then be useful for rehabilitation and strengthening purposes, and in providing a task in which loading can be controlled in examining metabolic responses to joint loading in human participants.

## Methods

### Participants

Participants were excluded if they reported a history of neurological or musculoskeletal disorders; or an injury, pain or surgery on their lower body or back in the six months prior to participation. Twenty healthy males (age 22.0 ± 2.8 years, height 1.72 ± 0.1 m, body mass 83.2 ± 14.9 kg; mean ± SD) were included. The local institutional research ethics board provided approval of the methods used in this study. The study was performed in accordance with the ethical standards of the Declaration of Helsinki. All participants provided informed consent prior to participation. A sample size calculation was based on preliminary data obtained with the first 10 participants; results indicated that a total of 20 participants would provide adequate statistical power (>80%).

### Setup and Protocol

Participants wore their own athletic shoes in order to improve ecological validity of the study. Infrared reflective markers were placed on the bony landmarks [25], to produce a 6-segment kinematic model: pelvis, left and right thigh, left and right shank, and left and right foot [26]. The three-dimensional kinematic model created using Visual3D software (v4.84.0, C-Motion Inc., Ontario) has been shown to produce results of good to excellent reliability during a similar drop-landing protocol [27]. All offline processing was conducted using Visual3D, which was successfully utilized in previously published works [28–31]. Marker movement was recorded using a 7-camera motion capture system (MX40, Vicon, Colorado). Marker position was sampled at a frequency of 100 Hz and filtered offline using a digital Butterworth 4th order low-pass filter with a 6 Hz cutoff. The filter cutoff frequency was determined using residual analysis of the difference between filtered and unfiltered signals [32] over 0.5–25 Hz range in 0.5 Hz steps with right ankle marker position data from a randomly selected trial recorded for the first four participants; the average cut-off frequency was used.

All trials were initiated with participants standing on an elevated platform at either of two heights: 22 cm (low) and 44 cm (high). The low height was consistent with the height of one riser for typical household stairs, while the high was consistent with the height of two risers. Participants were asked to step outwards off the platform with the rear leg straight and drop such that both feet simultaneously contacted two force plates (OR6-7, AMTI, Massachusetts), with one foot on each force plate. To standardize upper body movement between participants and conditions, participants were asked to lace fingers and maintain their hands over the abdomen during all trials. To avoid participants using different strategies when leaving the elevated platform, the investigator demonstrated the step-off technique and instructed participants explicitly to not jump off or lower themselves. Force plate signals were sampled at 1000 Hz and filtered offline using a Butterworth 4th order low-pass filter with an 8 Hz cutoff. The filter cutoff frequency was determined using a residual analysis approach [32] over 0.5–25 Hz range in 0.5 Hz steps with vertical ground reaction force (GRF) signal from a randomly selected trial recorded for the first four participants; the average cutoff frequency was used. A similar drop-landing methodology has been previously validated and utilized by Niehoff et al. [33] to examine joint loading in response to drop landing in a similar cohort.

Participants performed 10 drop-landing trials in each of six conditions, which were defined by combinations of two heights (“low”, “high”) and three instruction sets (“soft”, “natural” and “stiff”), for a total of 60 drop-landing trials. On average, participants performed one drop per minute per block of conditions. In addition, rest/recovery breaks were provided between each of the conditions to ameliorate the effects of fatigue. All participants completed the natural trials first, for both platform heights. For the natural instruction, participants were instructed to land on the force plates in whatever manner felt comfortable and natural–“land as naturally as possible”. The order of platform heights (low, high) was counterbalanced across the participants. Following the completion of the low and high trials under the natural instruction, all participants performed drop-landing trials under the soft and stiff instructions from the low and high heights (low-soft, low-stiff, high-soft, and high-stiff), which were arranged in random order. In the soft landing, participants were instructed to “land softly, absorbing the force at landing”; in the stiff landing, participants were instructed to “land stiffly, without absorbing the force at landing”. The investigator demonstrated the soft landing technique with exaggerated flexion at the knee and the stiff landing technique with reduced flexion at the knee. Participants reported that they replicated soft and stiff landings with exaggerated and reduced knee flexion as demonstrated.

### Measures of Interest

To confirm the change in the kinematics and kinetics of landing between drop-landing heights and instruction conditions, knee flexion angle and vertical GRF during landing were calculated. All measures of interest, except the knee angle, were normalized to participant’s body mass. Peak knee flexion angle and peak GRF were then reported.

To investigate the effect of different drop heights and landing instructions on knee joint loading the following measures were calculated bilaterally: knee joint compression force, knee joint flexion moment, knee joint abduction moment, and knee joint external rotation moment. Joint compression force was operationally defined as joint reaction force that acted along the longitudinal axis at the proximal end of the leg segment. The values for knee abduction and external rotation moment were reported at the peak knee flexion moment, in order to aid the estimation of the knee joint soft tissue injury risk. In this study, net external moments, which represent the external load on the joint, were described. Ankle, knee, and hip joint power as well as ankle and hip sagittal moment were calculated bilaterally, and the values of each were reported at the time of peak knee flexion moment to describe neuromuscular control during landing.

The measures of interest were averaged across the lower limbs for each trial following the analysis which confirmed no bilateral differences. All measures were calculated in Visual3D using the validated marker set reported earlier, and three-dimensional inverse dynamics algorithms [27, 34]; and resolved into the proximal segment coordinate system. All inertial segment properties were estimated from anthropometric data as per Dempster et al. [35].

### Statistical Analysis

All statistical analyses were conducted using JMP (v8.0, SAS Institute, North Carolina). A two-factor (*height* [low/high] X *instruction* [soft/natural/stiff]) mixed effects (*participant*–random effect, *height* and *instruction*–fixed effect) repeated measures analysis of variance (rmANOVA) was used to test for differences in the dependent measures between drop-landing conditions. Contrast analyses with Tukey HSD correction were performed to compare means and test interactions.

Fatigue can affect measures obtained using the inverse dynamics approach resulting in the order effect. To examine the potential order effect on knee joint compression and flexion moment, the first three consecutive trials of the third condition were compared with the last three consecutive trials for the sixth condition, across the low-soft, low-stiff, high-soft, and high-stiff condition blocks. A one-way (*order* [first/last]) mixed effects rmANOVA (*order*–fixed effect, *participant*–random effect) was conducted separately for each main measure of interest. The effect size was reported using generalized eta squared $\left({}_G^2\right)$ and considered trivial (<0.02), small (0.2–0.12), moderate (0.13–0.25), and large (≥0.26) [36].

## Results

### The Analysis of the Trial/Condition Order Effect

There was no significant effect of order for the peak knee compression force or the peak knee flexion moment.

### Peak Kinematic and Kinetic Measures

#### Knee Flexion Angle

There was no significant interaction effect, but the main effects of height and instruction were significant (Table 1). Peak knee flexion angle was larger for the high drop height than for the low. Peak knee flexion angle was greater under the soft instruction than under both natural and stiff instruction; peak knee flexion angle under the natural instruction was greater than under the stiff (Table 2).

**Table 1:** Summary of the statistical analyses

Peak measures	Interaction df(2,38)				Height *df*(1,19)				Instruction *df*(2,38)			
	*F*	*p* value	Effect size		*F*	*p* value	Effect size		*F*	*p* value	Effect size	
Knee flexion angle (°)	1.6	0.21	0.01	T	206.2	<0.01	0.30	L	103.0	<0.01	0.57	L
Vertical nGRF (N/kg)	0.5	0.64	<0.01	T	104.8	<0.01	0.27	L	86.8	<0.01	0.53	L
Knee compression force (N/kg)	0.1	0.89	<0.01	T	68.0	<0.01	0.17	M	90.8	<0.01	0.53	L
Knee flexion moment (Nm/kg)	16.5	<0.01	0.05	S	230.2	<0.01	0.45	L	15.8	<0.01	0.05	S
Measures at the peak knee flexion moment												
Knee abduction moment (Nm/kg)	2.4	0.10	0.01	T	44.3	<0.01	0.14	M	0.2	0.82	<0.01	T
Knee external rotation moment (Nm/kg)	2.2	0.13	0.01	T	14.7	<0.01	0.06	S	5.8	<0.01	0.02	T
Ankle flexion moment (Nm/kg)	2.6	0.08	0.01	T	105.1	<0.01	0.37	L	0.5	0.60	0.01	T
Hip extension moment (Nm/kg)	1.1	0.36	<0.01	T	1.7	0.21	0.01	T	6.5	<0.01	0.05	S
Ankle power (W/kg)	5.3	0.01	0.03	S	85.7	<0.01	0.48	L	3.3	<0.05	0.05	S
Knee power (W/kg)	9.4	<0.01	0.03	S	139.3	<0.01	0.63	L	7.0	<0.01	0.06	S
Hip power (W/kg)	10.3	<0.01	0.06	S	2.8	0.11	0.04	S	12.2	<0.01	0.07	S
Measures of trial order effect	Order df(1,19)											
	*F*	*p* value	Effect size									
Peak knee compression force (N/kg)	0.2	0.701	<0.01	T								
Peak knee flexion moment (Nm/kg)	0.56	0.465	0.01	T								

The effect sizes are reported using generalized eta squared. *T* trivial, *S* small, *M* moderate, *L* large

**Table 2:** Summary of the results which were not included in the figures

Peak measures	Low						High					
	Soft		Natural		Stiff		Soft		Natural		Stiff	
Knee flexion angle (°)	75.49	(2.39)	51.31	(2.75)	45.17	(1.95)	87.71	(2.70)	67.26	(2.88)	59.17	(1.71)
Vertical nGRF (N/kg)	12.71	(0.32)	18.18	(0.78)	22.36	(1.03)	17.24	(0.66)	23.05	(0.99)	26.60	(1.06)
Measures at the peak knee flexion moment												
Knee abduction moment (Nm/kg)	0.52	(0.29)	0.47	(0.26)	0.44	(0.34)	0.7	(0.36)	0.76	(0.39)	0.75	(0.42)
Knee external rotation moment (Nm/kg)	0.04	(0.07)	0.07	(0.12)	0.05	(0.12)	−0.05	(0.14)	−0.02	(0.14)	0.02	(0.17)
Ankle flexion moment (Nm/kg)	0.88	(0.04)	0.83	(0.05)	0.74	(0.11)	1.19	(0.06)	1.21	(0.06)	1.16	(0.07)
Hip extension moment (Nm/kg)	0.18	(0.11)	0.41	(0.14)	0.56	(0.20)	0.09	(0.12)	0.35	(0.20)	0.56	(0.22)

Mean (SE)

#### Vertical nGRF

There was no significant interaction effect, but the main effects of height and instruction were significant (Table 1). Peak ground reaction force (nGRF) was larger for the high drop height than for the low. Peak nGRF increased with instruction to land stiffly, from soft to natural to stiff (Table 2).

#### Knee Compression Force

**Fig. 1:**
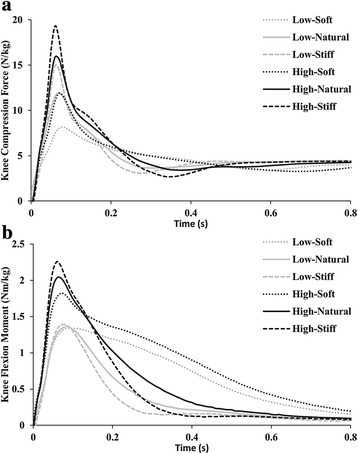
Averaged (*n* = 20) time series for the body mass-normalized knee joint compression force (**a**) and flexion moment (**b**) as a result of a drop from both low and high heights. The compression force peaks for both drop heights and all three instructions are temporally aligned at approximately 0.07 s. The flexion moment peaks are temporally aligned at approximately 0.07 s for the low-stiff, high-soft, high-natural, and high-stiff conditions; while the peaks for the low-soft and low-natural conditions are temporally aligned at approximately 0.09 s

**Fig. 2:**
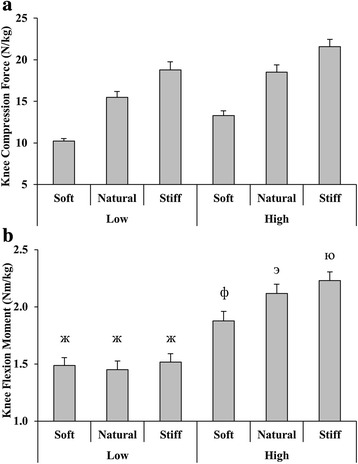
**a** Peak normalized knee joint compression force and **b** peak normalized external knee joint flexion moment. The *error bars* are SE. **a** Both main effects of height and instruction were significant. **b** Significant interaction effect between height and instruction was observed. Levels not connected by the same symbol are significantly different

There was no interaction effect, but the main effects for height and instruction were significant (Table 1). Peak knee compression force was larger for the high drop height than for the low. Peak compression knee force increased with instruction to land stiffly, from soft to natural to stiff (Figs. 1a and 2a).

#### Knee Flexion Moment

There was a significant interaction effect observed (Table 1). Peak knee flexion moment was not different between the low-soft, low-natural, and low-stiff conditions. Peak knee flexion moment in the high-soft condition was larger than in the low-soft, low-natural, and low-stiff condition, but lower than in the high-natural and high-stiff condition, which were not different from each other (Figs. 1b and 2b).

### Measures Reported at the Peak Knee Flexion Moment

#### Knee Abduction Moment

There was no significant interaction effect or main effect of instruction; however, the main effect of height was significant (Table 1). Knee abduction moment was larger for the high drop height than for the low (Table 2).

#### Knee External Rotation Moment

There was no significant interaction effect, but the main effects for height and instruction were significant (Table 1). Knee external rotation moment was larger for the low drop height than for the high. Knee external rotation moment was lower under the soft instruction than under both natural and stiff, which were not different from each other (Table 2).

#### Ankle Flexion Moment

There was no significant interaction effect or main effect of instruction; however, the main effect of height was significant (Table 1). Ankle flexion moment was larger for the high drop height than for the low (Table 2).

#### Hip Extension Moment

There was no significant interaction effect or main effect of height; however, the main effect of instruction was significant (Table 1). Hip extension moment was smaller for the soft condition than for the natural and stiff, which were not different from each other (Table 2).

#### Ankle Power

**Fig. 3:**
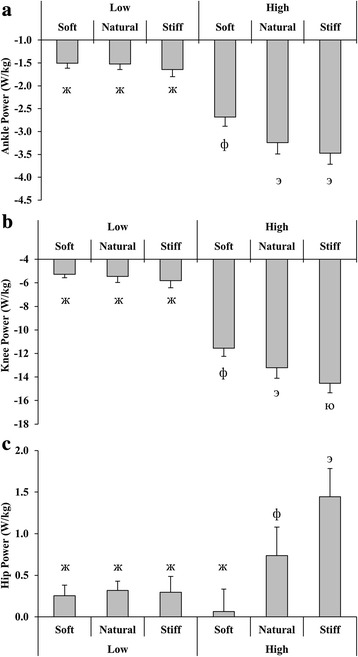
Normalized joint power measured at the peak knee joint flexion moment for **a** ankle, **b** knee, and **c** hip. The *error bars* are SE. Significant interaction effects between height and instruction were observed in **a**–**c**. Levels not connected by the same symbol are significantly different. The negative power represents energy absorption and eccentric activation. The positive power represents energy generation and concentric activation

There was a significant interaction effect observed (Table 1). Ankle negative power (energy absorption) was not different between instructions for the low height. Ankle negative power was greater for the high height for all instructions and increased from soft to natural and stiff, which were not different (Fig. 3a).

#### Knee Power

There was a significant interaction effect observed (Table 1). Knee negative power was not different between instructions for the low height. Knee negative power was greater for the high height for all instructions and increased from soft to natural to stiff instruction (Fig. 3b).

#### Hip Power

There was a significant interaction effect observed (Table 1). Hip positive power (energy generation) was not different between instructions for the low height. Hip positive power was smaller in high-natural condition than in high-stiff, but larger than in high-soft and all of the low instruction conditions, which were not different from each other (Fig. 3c).

## Discussion

The current study suggested a drop-landing task that makes use of drop heights that may be appropriate for rehabilitation purposes and are functional in daily life. This study examined transient knee joint loads during the drop-landing response and the effect of drop height and instruction cues on knee joint loads. Further, this study took steps toward a development of standardized exercise task to examine the effect of acute bouts of mechanical loading on bone and cartilage metabolism in humans, since previous research has been focused on animal models [15, 16]. Consistent with previous literature [24, 30], we found that the knee joint compression force increased with drop height and the instruction to land stiffly. Though sagittal knee joint moment scaled with instruction at the high height, it did not change with instruction set at the low height, suggesting that while instruction can increase knee joint compression at the low height, knee joint moment and perhaps; therefore, joint injury risk are not increased with the instruction set at the low height. Precautions were taken to standardize drop-landing task; demonstrations of step-off and landing techniques were provided. The results suggest relatively low, approximately 7%, variability within subjects in the measure of peak sagittal knee angle across conditions (Table 2).

Knee compression force was modulated by instruction; Fig. 2a suggests that compression force can be reduced by approximately 50% if the soft instruction is used, or increased by approximately 30% if the stiff instruction is used, relative to the natural landing instruction. As compression force has been associated with promoting tissue remodeling [12], the exercise task we proposed could improve bone and joint health. Since increased joint moment is associated with ligament injury [8], the absence of increased knee moment suggests that individuals could perform drop-landing task from the low height as opposed to high height without incremental risk of joint injury.

### The Effect of Drop Height on Lower Limb Joint Kinetics and Kinematics

The increase in the intensity of the drop-landing task at the greater drop-height resulted in elevated knee flexion angle during landing, vertical GRF, and knee joint compression force. The current findings are in agreement with previous research, which has demonstrated greater peak knee flexion angle and peak vertical GRF when landing from 60 cm compared with 20 cm [37, 38]. The aforementioned biomechanical measures all increase to attenuate impact forces during landing phase. While average frontal and transverse plane moment, which were consistent with previously reported values [39, 40], were found to be statistically different between heights with moderate and small effect size, respectively, the values were smaller than those experienced by young adults in sport setting (abduction moment 1.3 Nm/kg and external rotation moment 0.2 Nm/kg) [41, 42]; and likely not clinically relevant.

### The Effect of Instruction on Lower Limb Joint Kinetics and Kinematics

The results revealed the scaling of vertical nGRF, knee joint flexion angle, and compression force with increased landing stiffness. Consistent with the current results, previous research has indicated that instruction to land softly, as opposed to stiffly, from 40 cm resulted in lower vertical nGRF and larger knee joint flexion angle [22]. Additionally, the magnitude and timing of the peak knee compression force (Fig. 1a) were comparable to previously published data [43]. Finally, the force profiles for instruction condition signals were qualitatively comparable between the low and high drop height (Fig. 1a), which was consistent with our expectations.

### The Effect of Drop Height and Instruction on Lower Limb Joint Kinetics and Kinematics

The increase in drop height and landing stiffness resulted in increased knee joint compression force. The compression force increased from soft to natural to stiff instruction for both low and high drop heights and was greater for the high drop height (Fig. 2a). Since the increase in landing stiffness produced a linear scaling of the ground reaction force and the knee compression force, it is reasonable to suspect that a similar pattern between drop height and landing stiffness would be observed in the knee flexion moment, i.e., the increase in the flexion moment from soft to natural to stiff for the low and high heights. However, this was not the case. While the instruction to increase landing stiffness from the high height did result in the increase of the knee flexion moment, there was no difference in the moment observed with this instruction during landing from the low height.

The lower limb power analysis revealed that at the peak knee flexion moment, both the ankle and the knee showed negative power (Fig. 3a and b) and flexion joint moments (Fig. 2b; Table 2), indicating energy absorption and eccentric activation of the associated musculature, which is consistent with previous research [23]. In contrast to previous reports [23], however, the current study revealed positive hip joint power (Fig. 3c) and extension moment (Table 2), indicating concentric activation rather than eccentric. The concentric activation about the hip likely served to rotate the trunk forward and bring the body’s center of mass closer to the knee joint center in order to reduce the flexion moment at the knee by decreasing the effective length of the moment arm. A shorter moment arm, given that the ground reaction force magnitude was unchanged, would produce a lesser flexion moment about the knee joint. Importantly, in the low height condition, all three joints showed no difference in power magnitude across all levels of instruction, which suggests an equal rate of energy transfer between levels of instruction observed for each of the three joints (Fig. 3). In contrast, at the high height, the rate of energy absorption (the ankle and knee; Fig. 3a and b) and energy generation (the hip; Fig. 3c) increased across instruction levels in all three joints. The difference in patterns between heights as seen across instruction levels in the knee flexion moment and power measures may be driven by the kinetic energy absorption demand. Kinetic energy absorption demand was larger during landing from the high height than from the low; hence, the instruction condition produced a more pronounced energy absorption response at the high drop height than at the low. Considering that the hip showed positive power and that there was a significant interaction effect between the drop height and instruction condition with positive hip power increasing across instruction levels at the high height, but not at the low, it appears as though neuromuscular control of the hip at the low height reduced the loading effect at the knee. However, despite the increase in the positive hip power at the high height, it appears, the capacity of the hip joint to attenuate or obviate the development of additional moment at the knee became relatively less pronounced, as the hip joint became unable to efficiently reduce knee joint flexion moment. The dissimilarity between the previously reported data and the current findings with respect to hip power is likely because previous studies often reported peak joint power, while the current paper reported joint power values measured at the peak knee flexion moment. The peak values of measures of interest may often be temporally misaligned with one another and the event of interest, which in this paper was defined as the peak knee flexion moment. Hence, in order to explain why the peak knee flexion moment did not change with instruction at the low height, when it has increased significantly at the high height, we investigated joint power measured specifically at the peak knee flexion moment.

### Clinical Implications

Studies agree that the majority of soft tissue knee injuries are non-contact and occur during sudden deceleration and/or landing maneuvers [19]. The literature suggests that loading through the quadriceps may be one of the mechanisms leading to knee joint ligament injury, as the quadriceps muscle activity producing sagittal moment has been shown to generate large shear force pulling tibia anteriorly on femur [20, 44]. In this study, knee compression forces as well as sagittal moments were larger in the high drop height. In the low height, however, while the knee compression force increased with instruction, the instruction did not influence the knee flexion moment. Since an increase in the knee flexion moment may increase the risk of joint injury, it appears that the knee joint compression force, and thus, perhaps tissue stimulation toward cartilage formation, during landing from a lower 22 cm height (consistent with a household stair) can be increased using an instruction to land more stiffly without suggestion of increase in the risk of knee joint injury as the knee flexion moment did not increase with landing stiffness.

### Limitations

The current study is limited by inclusion of male participants only. Future research should include female participants, as due to the anatomical differences, the kinetics and kinematics of the lower limb may differ between sexes. Further, the current study is limited by the use of an inverse dynamics approach. The authors recognize that the bone-on-bone force does not correspond directly to joint compression force calculated using inverse dynamics approach [32]. The inverse dynamics approach does not consider muscle activation force, which adds to the compression force in the knee. Previous research has shown comparable knee loads during stair climb to the current findings during the low-soft condition, while the tibiofemoral bone-on-bone force was 3.5-fold larger when compared to joint reaction force [45]. Nevertheless, the inverse dynamics approach is a feasible and simpler alternative to techniques used to quantify joint bone-on-bone loading [46]. The future work should include muscle force modeling to improve joint compression force estimation. Lastly, a relatively large number of trials collected in this study may have resulted in participant fatigue despite that participants received rest/recovery breaks. Fatigue can affect measures obtained using the inverse dynamics approach resulting in the order effect. We mitigated the effect of fatigue by counterbalancing the first two blocks of trials across participants and randomly assigning the last four trial blocks. With the follow-up analysis, we showed no difference between the earlier and later trial means which suggests that fatigue, if present, did not affect the measures of interest.

## Conclusions

The drop-landing task creates an increase in knee joint compression loading that can be scaled using specific instructions. Use of 22 cm height may be safer and beneficial for joint and bone injury rehabilitation, as it represents a height that is much lower than previously discussed in the literature, and is representative of a standard stair height and therefore functionally accessible. Our aim is to utilize the outcomes of the current study in future work aimed at establishing a standardized task to examine the effect of acute bouts of mechanical loading on bone and cartilage metabolism.
